# Adult Intestinal Intussusception Caused by the Gastrojejunostomy Tube: An Endoscopically Treatable Phenomenon

**DOI:** 10.1155/2021/4325443

**Published:** 2021-06-11

**Authors:** Kermit S. Zhang, Jash Bansal, Anmol Bansal, Vikas Chitnavis

**Affiliations:** ^1^Virginia Tech Carilion School of Medicine, Roanoke, VA, USA; ^2^Department of Gastroenterology, Carilion Clinic Memorial Hospital, Roanoke, VA, USA; ^3^Department of Radiology, Carilion Clinic Memorial Hospital, Roanoke, VA, USA

## Abstract

Adult duodenoduodenal intussusception is extremely rare due to the retroperitoneal fixation of the second, third, and fourth parts of the duodenum. A majority of clinically significant intussusception with identifiable etiologies is typically neoplastic with more rare causes including retained food and indwelling enteral tubes, specifically with gastrojejunostomy (GJ) tubes. Herein, we discuss the case of a 23-year-old male who developed duodenoduodenal intussusception upon a PEGJ placement with associated gastroduodenal dilation and telescope phenomenon. To the best of our knowledge, there are no reports of intussusception found to be caused by GJ tubes in the adult population. The reported patient was found to have a 4-cm enteroenteric intussusception without obstruction or ischemia with bowel thickening proximal to the pathology. Although adult intussusception cases are typically managed surgically, we were able to reduce the intussusception via endoscopy due to rapid diagnosis upon presentation and intervention before the bowel wall could be compromised.

## 1. Introduction

Intussusception is the invagination of a distal segment of the small intestine into the proximal segment. By itself, this is a common phenomenon often disregarded within highly mobile segments of the jejunum. This condition is extremely rare in the duodenum due to the retroperitoneal fixation of the second, third, and fourth parts of the duodenum. In 80–90% of cases of clinically significant intussusception whose etiologies are identified, the lead point is typically neoplastic; this includes lesions such as Brunner's gland hamartomas, carcinomas, lipomas, and adenomas [1]. Additional causes of adult intussusception are polyps, strictures, and idiopathic and colonic diverticulum. While majority of cases self-resolve, those that do not can suffer compromised vascular flow and subsequent necrosis. The development of enteroenteric intussusception associated with indwelling enteral tubes is an extremely rare occurrence and is associated with substantial morbidity and mortality [2, 3]. Herein, we discuss the case of a 23-year-old male who developed duodenoduodenal intussusception upon a percutaneous gastrojejunostomy (PEGJ) and was with associated gastroduodenal dilation and telescope phenomenon.

## 2. Case Presentation

A 23-year-old male was hospitalized with generalized abdominal pain, nausea, and vomiting for 3 days. His medical history was significant for a traumatic cervical spinal injury six months prior with consequent paraplegia. He has been dependent on tracheostomy with ventilation and PEGJ placement since.

Vital signs on admission included a heart rate of 146 bpm, respiratory rate of 17, temperature of 99.8 F, and blood pressure of 89/51 mmHg. He was alert and oriented, but chronically-ill appearing with dry mucous membranes. His abdominal examination demonstrated a distended, diffusely tender abdomen with guarding and hypoactive bowel sounds. There was a bloody exudate at the ostomy site. Pertinent labs included a white blood cell count of 14,000/*μ*L, hemoglobin of 17.3 g/dL, alkaline phosphatase 161 IU/L, and lactate of 0.6 mmol/L.

**Figure 1:**
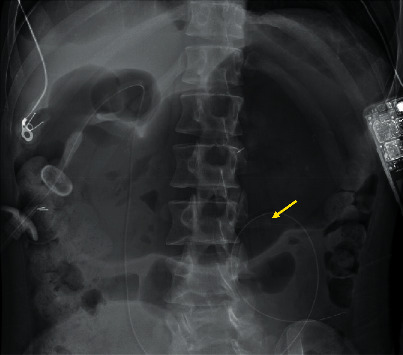
Abdominal X-ray demonstrating severe gaseous distention of the entire stomach with the balloon of presumed gastrostomy projecting over the right abdomen. The tip of a jejunostomy tube placed through the gastrostomy projects over the left midabdomen (yellow arrow). There is no gross evidence of free air noted given inherent limitations of this portable supine radiograph.

**Figure 2:**
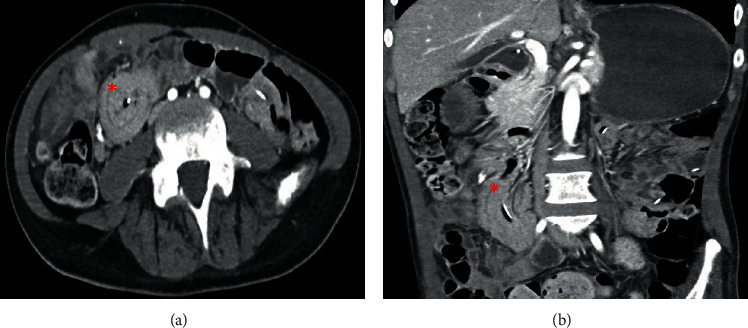
Axial and coronal computed tomography of the (a) abdomen and (b) and pelvis (with contrast) demonstrating enteroenteric intussusception at the junction of the second and third portions of the duodenum (red asterick) with the jejunostomy limb acting as the presumed lead point.

An abdominal plan radiograph revealed severe gaseous distension of the stomach and a large volume of stool in the colon (Figure 1). An abdominal computed tomography (CT) scan demonstrated the gastrojejunostomy (GJ) entering the gastric antrum, traversing the duodenum, and terminating in the midjejunum. A paucity of enteral fluid with a concentric soft tissue density surrounding the enteric catheter was shown at the junction of the second and third portions of the duodenum (Figure 2). This telescoping was interpreted by the radiologist as a 4 cm duodenoduodenal intussusception with the jejunostomy limb acting as a lead point. The jejunal and ileal loops appeared normal in caliber. There was no evidence of small bowel stranding, necrosis, ischemia, or perforation.

**Figure 3:**
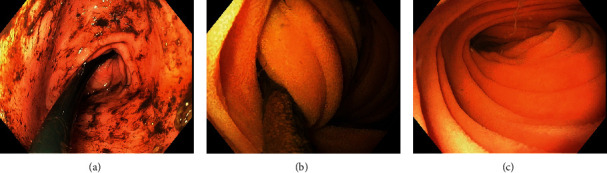
Esophagogastroduodenoscopy demonstrated retained semisolid food at the pyloric junction (a), significant bowel obstruction due to intussusception likely as a result of mucosal traction from the patient's jejunostomy limb tip at the second portion of the duodenum (b), and subsequent patent bowel following reduction in the intussuscepted bowel following reduction (c). The jejunostomy was also removed being replaced with a gastrostomy tube.

The gastroenterology service was consulted and planned for immediate reduction of the intussusception by removing the GJ. The endoscopy confirmed that the jejunostomy tube held traction on a segment of the distal bowel that had invaginated into the second portion of the duodenum (Figure 3). The tip of the endoscope was used to prevent the distal bowel from traveling proximally while the GJ removed and subsequently replaced with the gastrostomy tube. Postprocedural recovery was uncomplicated, and the patient's leukocytosis and lactate levels returned to the normal range within 24 hours.

## 3. Discussion

This report presents an adult case of duodenoduodenal intussusception and telescope phenomenon across the duodenal flexure caused by a GJ. There have been reports of cases in which a gastrostomy tube caused intussusception at the duodenal site related to improper fixation of the tube coupled with normal peristalsis. To our knowledge, few reports of intussusception have been found that it is caused by GJ tubes amongst adults, and the underlying mechanism for intussusception is not definitively understood. One theory by Wu et al. hypothesized it may be due to the tip of a jejunostomy limb inducing an inflammatory response causing mucosal hypertrophy leading to the formation of a lead point [4]. Similar to Wu et al., we believe that the PEG tube may not have been adequately sterilized between use allowing for inflammation surrounding the jejunostomy tip resulting in mucosal hypertrophy leading to this complication.

The successful management of intussusception depends on a reasonable degree of suspicion, early diagnosis, and prompt reduction. Treatment modalities for intussusception are variable. Amongst the 90% of adult intussusception cases that do not resolve spontaneously, surgery is often the intervention of choice due to the high likelihood of an associated pathologic lead point [5]. This is commonly employed in patients with symptomatic bowel obstruction and signs concerning for malignancy such as anorexia, unintended weight loss, and night sweats. Adults with intussusception in the large intestine such as ileocolic and colocolic intussusception may also be candidates for surgery given the higher association for neoplasm [6]. Nonoperative management including endoscopic reduction and radiologic decompression are reasonable interventions amongst patients without such clinically severe features [3]. The current existing literature describes a high risk of perforation and recurrence after endoscopic intussusception reduction [7]. However, retrospective studies have demonstrated successful nonoperative and minimally invasive management in as many as 82% of radiographic intussusceptions even in the setting of gastrointestinal symptoms [6].

Given the nonspecific nature of the clinical presentation of intussusception in adults and the need to exclude other critical diagnoses, CT is the imaging modality of choice. Classic findings on CT include the “bulls-eye” or sausage-shaped concentric lesions created by the anatomic invagination of the affected enteric segments. Furthermore, CT can identify compromised vascular perfusion of the mesenteric vessels within the bowel lumen. Carucci et al. reported a series of four cases of jejunojejunal intussusception following a jejunostomy feeding tube with distinguishable radiographic findings while the feeding tube was still in place [8]. However, radiography has its diagnostic limitations as Kareem et al. reported a case of adult jejunojejunal intussusception secondary to GJ placement with no distinguishable findings on CT [9]. The findings in our patient's CT clearly demonstrated a telescope phenomenon surrounding the jejunostomy limb and were amenable to endoscopic reduction.

In our case, factors that contributed to successful endoscopic management were rapid diagnosis and intervention before bowel integrity could be compromised. This allowed for optimal conditions to treat the patient without undue burden of an operation in traditionally surgically managed patients as there was low concern for malignancy based on patient presentation and radiography. In an era of advancing endoscopy and a focus on minimally invasive procedures, adult intussusception without complications can be considered for endoscopic management.

## Data Availability

The data used to support the findings of this case are available from the corresponding author upon request.
